# Automated Prognosis Marker Assessment in Breast Cancers Using BLEACH&STAIN Multiplexed Immunohistochemistry

**DOI:** 10.3390/biomedicines11123175

**Published:** 2023-11-29

**Authors:** Tim Mandelkow, Elena Bady, Magalie C. J. Lurati, Jonas B. Raedler, Jan H. Müller, Zhihao Huang, Eik Vettorazzi, Maximilian Lennartz, Till S. Clauditz, Patrick Lebok, Lisa Steinhilper, Linn Woelber, Guido Sauter, Enikö Berkes, Simon Bühler, Peter Paluchowski, Uwe Heilenkötter, Volkmar Müller, Barbara Schmalfeldt, Albert von der Assen, Frank Jacobsen, Till Krech, Rainer H. Krech, Ronald Simon, Christian Bernreuther, Stefan Steurer, Eike Burandt, Niclas C. Blessin

**Affiliations:** 1 Institute of Pathology, University Medical Center Hamburg-Eppendorf, 20246 Hamburg, Germany; 2 College of Arts and Sciences, Boston University, Boston, MA 02215, USA; 3 Department of Medical Biometry and Epidemiology, University Medical Center Hamburg-Eppendorf, 20246 Hamburg, Germany; 4 Institute of Pathology, Clinical Center Osnabrück, 49076 Osnabrück, Germany; 5 Department of Gynecology, University Medical Center Hamburg-Eppendorf, 20246 Hamburg, Germany; 6 Department of Gynecology, Albertinen Clinic Schnelsen, 22457 Hamburg, Germany; 7 Department of Gynecology, Amalie Sieveking Clinic, 22359 Hamburg, Germany; 8 Department of Gynecology, Regio Clinic Pinneberg, 25421 Pinneberg, Germany; 9 Department of Gynecology, Clinical Centre Itzehoe, 25524 Itzehoe, Germany; 10 Breast Center Osnabrück, 49076 Osnabrück, Germany

**Keywords:** breast cancer, prognosis markers, multiplex fluorescence immunohistochemistry, artificial intelligence

## Abstract

Prognostic markers in routine clinical management of breast cancer are often assessed using RNA-based multi-gene panels that depend on fluctuating tumor purity. Multiplex fluorescence immunohistochemistry (mfIHC) holds the potential for an improved risk assessment. To enable automated prognosis marker detection (i.e., progesterone receptor [PR], estrogen receptor [ER], androgen receptor [AR], GATA3, TROP2, HER2, PD-L1, Ki67, TOP2A), a framework for automated breast cancer identification was developed and validated involving thirteen different artificial intelligence analysis steps and an algorithm for cell distance analysis using 11+1-marker-BLEACH&STAIN-mfIHC staining in 1404 invasive breast cancers of no special type (NST). The framework for automated breast cancer detection discriminated normal glands from malignant glands with an accuracy of 98.4%. This approach identified that five (PR, ER, AR, GATA3, PD-L1) of nine biomarkers were associated with prolonged overall survival (*p* ≤ 0.0095 each) and two of these (PR, AR) were found to be independent risk factors in multivariate analysis (*p* ≤ 0.0151 each). The combined assessment of PR-ER-AR-GATA3-PD-L1 as a five-marker prognosis score showed strong prognostic relevance (*p* < 0.0001) and was an independent risk factor in multivariate analysis (*p* = 0.0034). Automated breast cancer detection in combination with an artificial intelligence-based analysis of mfIHC enables a rapid and reliable analysis of multiple prognostic parameters. The strict limitation of the analysis to malignant cells excludes the impact of fluctuating tumor purity on assay precision.

## 1. Introduction

Breast cancer is the most prevalent cancer in females worldwide [1]. Although adjuvant systemic therapy substantially improves patient survival [2,3], a more accurate risk assessment in breast cancer could further advance the selection of patients for adjuvant systemic therapy [4,5]. Classical prognostic parameters such as ER, PR, Ki67, nodal involvement, tumor size, and tumor grade [6,7,8,9] are often complemented in routine clinical practice by multi-gene RNA assays such as Endopredict or Oncotype DX [10,11]. These assays quantify the RNA expression level of up to 70 genes involved in cell cycle, angiogenesis, signal transduction, and other pivotal cell functions [12,13]. The main limitation of these RNA-based assays is the inherent contamination of cancer tissue by an unknown and variable fraction of non-neoplastic cells with a variable composition of benign epithelial, immune, or stroma cells [14]. Such contaminations can lead to false positive and negative results especially in breast cancers with abundant stroma and intermingled benign glands [14,15,16,17,18].

A cell type specific assessment of multiple molecular markers on breast cancer tissue sections represents an intuitive solution to address the issue of variable tumor purity. This is possible by using multiplexed immunohistochemistry. Although currently available multiplex fluorescence immunohistochemistry (mfIHC) approaches are often limited to six markers or a small tissue size that hampers translational studies on large tissue microarray cohorts, more recent mfIHC methods such as CODEX or BLEACH&STAIN have consistently increased the number of possible proteins to more than 20 biomarkers that can be stained on a single tissue section [19,20]. These methods enable staining of a plethora of prognosis markers, minimizing the size and amount of tissue that is needed from a biopsy. However, an algorithm for differentiating benign from malignant epithelial breast cells, i.e., automated breast cancer detection that is mandatory for large scale biomarker analysis in routine clinical practice, is still lacking.

In this study, we utilized mfIHC to develop a framework for automated breast cancer assessment premised on a recently published approach for automated prostate cancer detection and we evaluated its utility in a combined analysis of nine potential prognostic features.

## 2. Materials and Methods

Patients and tissues. The study included four tissue microarrays with tissue spots measuring 0.6 mm in diameter that were made from a total of 1530 primary tumors from breast cancers of no special type (NST) operated on between 2003 and 2012 at the Academic Hospital Fuerth and Clinical Center Osnabrueck (for exclusion criteria see technical aspects of the results section). Detailed histopathological data on grade, pathological tumor stage (pT), pathological lymph node status (pN), and pathological metastases status were available from up to 1522 tumors. Clinical follow-up data were available from 627 breast cancers operated on between 2007 and 2012 with a median follow-up time of 49 months (95% CI 46–49). Manual progesterone (PR) [21], estrogen receptor (ER), GATA3 [22], HER2, and PD-L1 [23] quantification using conventional brightfield IHC were available. All samples were from the archives of the Institutes of Pathology Fuerth and Clinical Center Osnabrueck (Germany). The use of archived remnants of diagnostic tissues for the manufacturing of TMAs and their analysis for research purposes, as well as patient data analysis, has been approved by local laws (HmbKHG, §12) and by the local ethics committee (Ethics commission Hamburg, WF-049/09, 25 January 2010). All work has been carried out in compliance with the Helsinki Declaration. (For patient characteristics, see Table S1 in the Supplementary Materials).

BLEACH&STAIN multiplex fluorescence immunohistochemistry (IHC). Multiplex fluorescence immunohistochemistry was performed using BLEACH&STAIN as previously described [20]. The 11 + 1 marker BLEACH&STAIN mfIHC was conducted in four sequential staining and imaging cycles of three biomarkers counterstained with DAPI and a bleaching step in between. In brief, freshly cut 4 µm consecutive tissue sections on X-tra^®^ glass slides (Cat. #3800204AE, Leica, Wetzlar, Germany) were initially boiled in an autoclave (30 min at 100–120 °C) for antigen retrieval. The bleaching step between every sequential 3 + 1 marker staining included photobleaching (metal halide lamp with 1600 W) combined with slide incubation in 3% hydrogen peroxide for chemical inactivation of fluorochromes and cooling (4 °C) of the slides during the bleaching process. Thus, 11 staining rounds of individual markers using OPAL fluorochromes (AKOYA, Marlborough, MA, USA), 10 times removal of the bound antibodies via a short microwave treatment (5 min at 100 °C and 5 min at 93 °C), 4 scanning rounds using the Leica Aperio VERSA 8, and, finally, alignment of the digital images using an artificial intelligence-based custom software written in Python version 3.8 were performed. Details on the used primary antibodies, antibody retrieval procedures, and fluorescence OPAL dyes are given in Table S2. 

Deep learning-based framework for automated 11 + 1-plex BLEACH&STAIN mfIHC image analysis. Image analysis was performed using the previously trained [24] deep learning-based (U-Net) framework for cell detection, cell segmentation, intensity measurement of the used fluorophores (range 0–255, i.e., a continuous numerical value indicating the fluorescence signal strength), processing the intensity values of 11 markers+ DAPI, and cell-to-cell distance analysis using Python version 3.8 [25], R version 3.6.1 (The R foundation, Vienna, Austria) [26] and the Visiopharm software package version 2020.08 (Hoersholm, Denmark). The intensity of each fluorochrome was recorded as raw intensity for the individual cells and normalized by the mean PanCK intensity on PanCK^+^ cells. Additionally, marker positivity (i.e., PR, ER, androgen receptor [AR], GATA3, TROP2, HER2, PD-L1, Ki67, TOP2A, Myosin, and PanCK) was evaluated via 11 U-Net systems as described previously [20,24]. 

**Figure 1:**
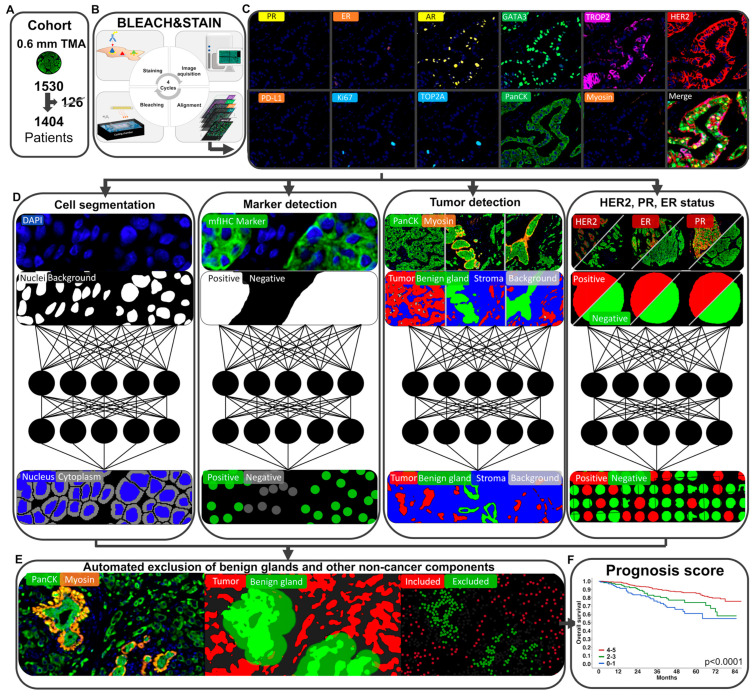
Framework for automated prognosis marker assessment in breast cancer. (**A**–**C**) The BLEACH&STAIN multiplex fluorescence immunohistochemistry technology facilitates high throughput staining of 11 biomarkers on 1404 invasive breast cancers of no special type (NST). (**D**–**F**) A deep learning-based framework for automated breast cancer detection, comprising 16 deep learning systems, was used to enable automated prognosis markers assessment (schematic illustrations of the neural networks are shown in (**D**)).

**Figure 2:**
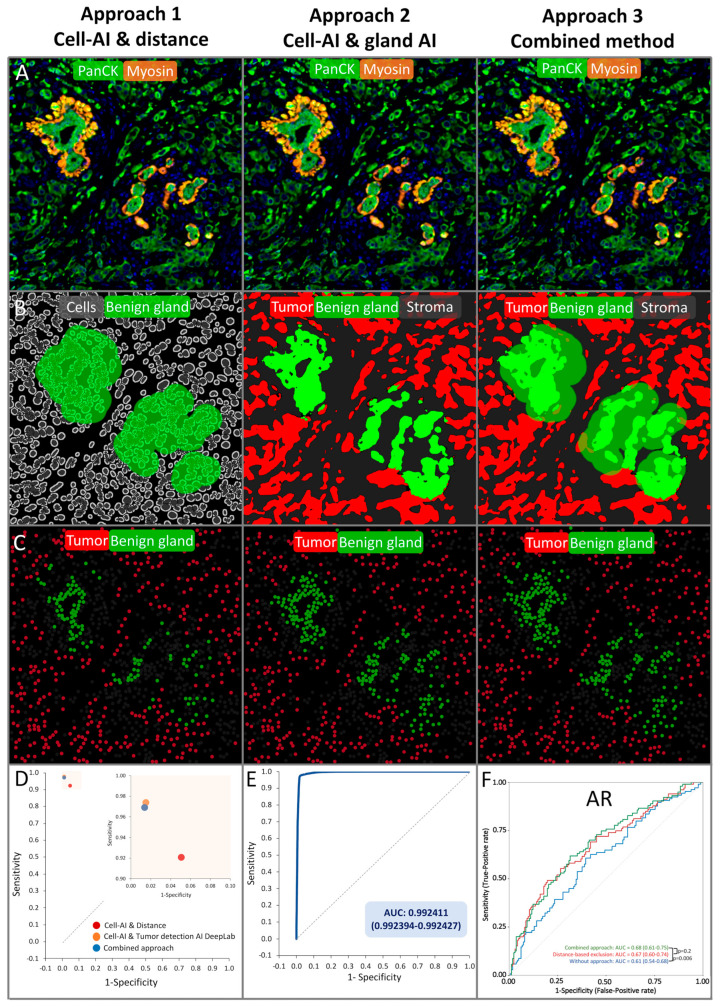
Approaches for automated breast cancer detection. (**A**–**C**) Approach 1 (Deep learning-based cell detection combined with distance analysis), approach 2 (“stand alone” gland detection deep learning system), and approach 3 (combination of approach 1 and 2) are shown by visualizing the classification performance in raw images (**A**,**B**) and the corresponding visualization of the calcification based on the output data (**C**). (**D**,**E**) Both approach 1 and approach 2 showed a sensitivity and specificity ≥ 0.9. (**F**) The combined approach 3 performed significantly better in time-depended receiver operating characteristic curves than the raw marker expression (*p* = 0.006).

Automated breast cancer detection. Invasive breast cancer cells can be differentiated from benign breast glands through the absence of the myoepithelial cell layer, similarly to invasive prostate cancer that lacks a basal cell layer. Therefore, a framework for automated breast cancer detection was structurally based on a prior model for prostate cancer detection (patent #WO 2023/285518) [24] and has been retrained for the automated detection of breast cancer (Figure 1). Thus, an algorithm to detect epithelial cells (i.e., PanCK^+^ cells) that were located ≤25 µm adjacent to myosin^+^ myoepithelial cells was combined with a deep-learning system for the detection of benign breast glands to differentiate between benign and malignant epithelial cells. This framework enabled the exclusion of benign breast glands, stroma cells, immune cells, and other non-cancer components to analyze biomarker expression exclusively on breast cancer cells. The optimal distance of ≤25 µm between tumor cells and myosin^+^ myoepithelial cells was evaluated in a test set of 374 annotations of benign and malignant breast epithelium (containing 98,264 benign and 99,229 malignant cells, Figure S1). The DeepLabv3+-based benign gland detection was trained and validated on 180 breast cancer patients (Figure 2, Table S3). The classification performances of cell–cell distance analysis, the DeepLabv3+ convolutional network, and the combination of both approaches were tested on the validation set (*n* = 613 glands), showing an accuracy of 98.4% for the combined approach (Figure 2, Table S3).

Marker pattern detection. Three DeepLabv3+ convolutional networks were trained for the detection of HER2^+^, ER^+^, and PR^+^ patients using 1048 tumor samples. Classification performances of the three DeepLab3+ convolutional networks were tested on the validation set (n = 356), showing an accuracy of 96.6% for the detection of HER2^+^, 96.4% for ER^+^, and 96.4% for PR^+^ patients (Figure S3C, Table S4). 

mfIHC score. The mfIHC score was calculated by the sum of the min-max normalized (range 0 to 50) proportion of marker-positive cells and the min-max normalized (range 0 to 50) mean intensity of marker-positive cells for each patient (Figures S2 and S3) following the immunoreactive score of Remmele and Stegner [27] as well as the Allred score [28]. 

Prognosis score based on the mfIHC score. A 5-marker prognosis score (PR, ER, AR, GATA3, and PD-L1) was formed (summation) based on whether a patient was in the high (1 point) or low groups (0 points) in a univariate analysis that was based on marker expression level (mfIHC score). The threshold for positivity for a marker in the univariate analysis was set at the point of the highest slope (and visually corrected) in the distribution plots (Figure S2C). The deep learning system for marker pattern detection was highly concordant with the mfIHC-based categorization in high and low groups (Figure S3C).

Statistical analysis. Statistical calculations were performed with R version 3.6.1 (The R foundation) [26,29] and JMP Pro 15 software package (SAS Institute Inc., Cary, NC, USA) [30]. Contingency tables and the chi² test were used to search for associations between molecular parameters and tumor phenotype. Survival curves were calculated according to Kaplan–Meier. The log-rank test was applied to detect significant survival differences between groups. Cox proportional hazards regression analysis was performed to test the statistical independence and significance between pathological, molecular, and clinical variables. Time-dependent areas under receiver operating characteristic curves were used to estimate the prognostic performance of the automated prognosis marker analysis (R “riskRegression” [31] package). Unsupervised cluster analysis using custom R scripts based on “gplots,” and “hclust,”, and unsupervised X-shift clustering [32] were applied to differentiate patient subgroups based on their marker expression pattern and MCL clustering was applied to investigate the relationship between the markers. All *p*-values were two-sided, and *p*-values < 0.05 were considered as significant).

## 3. Results

Technical aspects. A total of 1404 (92%) of 1530 samples of breast cancers of no special type (NST) were successfully analyzed in this study. The remaining 126 tumor samples were excluded due to the lack of tissue or representative cancer cells. The combine approach of both the distance-based and the DeepLabv3+-based breast cancer detection showed an accuracy of 98.4% (95% confidence interval [CI]: 97.4–99.3) and was thus used in this study (Figure 1 and Figure 2, Table S3). Receiver operating characteristic curves (ROC) confirmed a significantly improved prognostic performance of PR, AR, and GATA3 expression quantified by our approach for automated breast cancer detection compared to an assessment of these markers without using the approach (each *p* < 0.0197, Figure 2F and Figure S4). In addition, a comparison of the fully automated marker assessment in this study with manually assessed data using conventional brightfield immunohistochemistry from previous studies showed a high concordance for PR, ER, GATA3, HER2, and PD-L1 assessment (each *p* < 0.0001, Figure S5).

**Figure 3:**
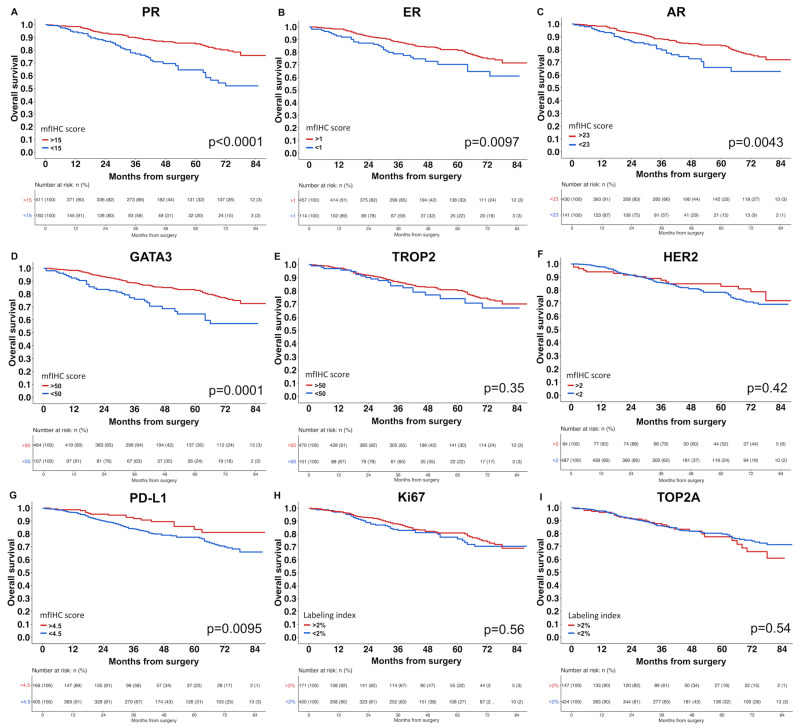
(**A**–**I**) Kaplan–Meier estimates for overall survival in breast cancer of no special type.

Breast cancer prognosis. A high expression level (mfIHC score) of PR, AR, GATA3, and TROP2 was significantly linked to low pT stage and low tumor grade (*p* ≤ 0.0002 each, Table 1). Accordingly, high expression of PR, ER, AR, GATA3, and PD-L1 was also associated with prolonged overall survival in the univariate analysis (*p* ≤ 0.0095 each, Figure 3). In contrast, high levels of HER2, Ki67, and TOP2A were linked to high tumor grade (*p* < 0.0001 each, Table 1), but they were unrelated to overall survival (Figure 3). In a multivariate analysis including pT, pN, and tumor grade, a high expression level of PR (mfIHC score > 15 vs. <15, HR 0.55, *p* = 0.0149) and AR (mfIHC score > 23 vs. <23, HR 0.54, *p* = 0.0151) expression were independent predictors of overall survival (Table S5).

**Figure 4:**
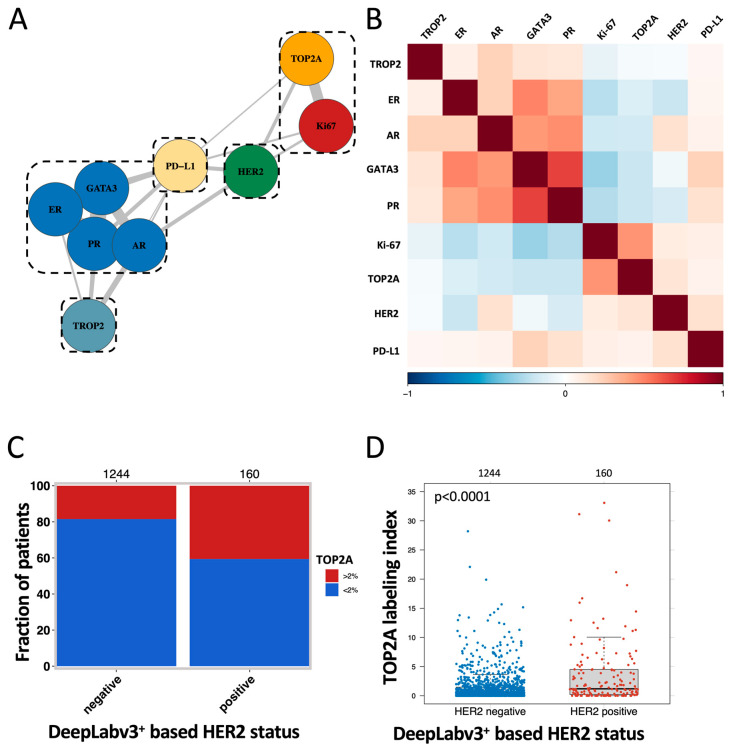
Unsupervised cluster analysis and coherence of HER2 and TOP2A expression. (**A**) Unsupervised MCL cluster of the analyzed prognostic markers. (**B**) Correlation heatmap of the analyzed prognostic marker. (**C**) Association of TOP2A-positive patients with the DeepLabv3+-based HER2 status (*p* < 0.0001). (**D**) Association of TOP2A labeling index with the DeepLabv3+-based HER2 status.

**Figure 5:**
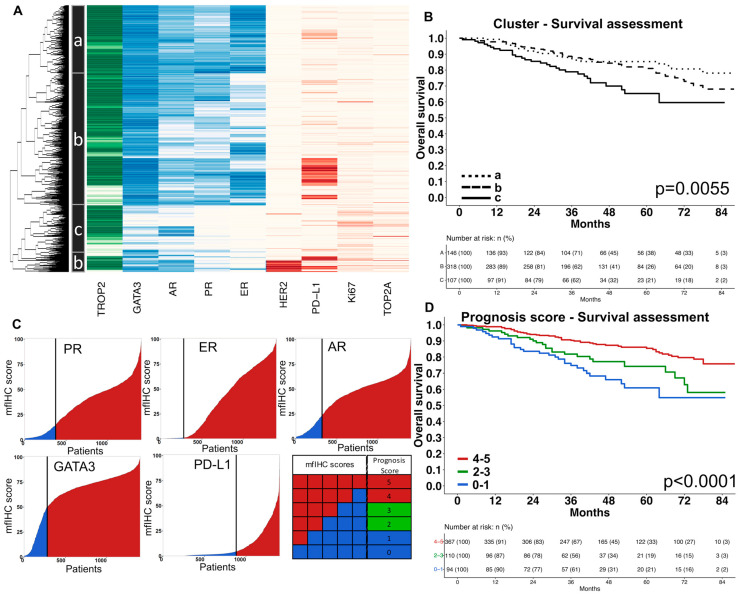
Combined marker assessment as prognosis scores. (**A**) Unsupervised cluster analysis of nine biomarkers revealed three clusters (a–c) with distinct expression patterns. (**B**) The Kaplan–Meier estimate of the three clusters based on the unsupervised cluster analysis. (**C**) The five-marker prognosis score was calculated as a numeric score for each patient based on the expression of the progesterone receptor (PR), estrogen receptor (ER), androgen receptor (AR), GATA3, and PD-L1. For each marker that was highly expressed (high mfIHC score), one point was added and results were recorded in a five-marker prognosis score (ranging from 0 to 5). (**D**) The Kaplan–Meier estimate showed strong prognostic impact of the five-marker prognosis score.

Marker interplay and prognosis scores. MCL clustering, t-SNE, and the Spearman’s correlation analysis revealed a strong link between PR, ER, AR, and GATA3 as well as between the proliferation markers TOP2A and Ki67 (Figure 4A,B and Figure S6). Accordingly, unsupervised hierarchical clustering revealed that the risk for a reduced overall survival increased continuously (Cluster a, b, c) along with the loss of PR, ER, AR, and GATA3 expression (Figure 5A,B and Figure S5). The combined assessment of PR, ER, AR, GATA3, and PD-L1—as a manually predefined five-marker prognosis score—showed strong prognostic relevance (*p* < 0.0001, Figure 5C) and was an independent risk factor (*p* = 0.0034) in a multivariate analysis including pT, pN, and tumor grade. A high TOP2A labeling index was significantly linked to HER2 positivity (*p* < 0.0001, Figure 4C) and three out of four patients with the highest TOP2A labeling index were also HER2 positive (Figure 4D).

## 4. Discussion

In this study, a framework for automated breast cancer assessment was developed based on our recently published approach for automated prostate cancer detection to investigate the prognostic relevance of nine different prognosis markers in breast cancer of no special type (NST) using 11 marker BLEACH&STAIN multiplex fluorescence immunohistochemistry. 

Approach and mfIHC score. A key feature of our approach was an automated tumor cell detection using a combination of pan cytokeratin (PanCK) for the detection of epithelial cells and a myoepithelial cell marker (Myosin H11) for exclusion of adjacent normal epithelial cells. Thus, this approach mimicked the way pathologists operate in routine clinical practice in order to identify breast cancer cells that are not accompanied by myoepithelial cells. Such an automated breast cancer identification approach facilitates the quantification of various prognosis markers in tumor cells for which both the fraction (number of positive tumor cells divided by all tumor cells) and the intensity level (fluorescence intensity as a surrogate for protein expression level) were assessed. Given that both the number of marker-positive tumor cells and the protein expression level drives the prognostic relevance, the mfIHC score was calculated and used in this study. Involving both parameters is in line with the immunoreactive score as described by Remmele and Stegner et al. [27] as well as the Allred score which is still used in routine breast pathology [28]. Several studies have shown that computerization of cumbersome manual quantification can improve the quality of a biomarker assessment and thus increase the predictive performance of prognosis markers [24,33]. Accordingly, several other prognostic systems that incorporate artificial intelligence-based analyses of H&E morphology and/or clinicopathological data have been shown to enhance the prognostic prediction of breast cancer [34,35].

Validation by prognostic relevance. Indirect validation of our approach was provided by the significant associations between high levels of progesterone receptor (PR), estrogen receptor (ER), androgen receptor (AR), GATA3 expression, and favorable tumor features as well as prolonged overall survival. High levels of PR, ER, and AR expression are well-established markers for a favorable prognosis of breast cancer patients [36,37]. The fact that GATA3 expression constituted a strong prognostic feature in our study was also in line with data from several recent studies which also suggested a strong link between reduced GATA3 expression and poor survival in breast cancer patients [22,38]. 

Ki67 and HER2. It is of note that a prognostic role was not seen for the Ki67 labeling index and HER2 expression in this study. Both HER2 overexpression/amplification [39,40] and high Ki67 labeling index (LI) [41] had been found to be strongly associated with poor patient survival in earlier studies. That both parameters are features of aggressive breast cancer is supported by their strong associations with high tumor grade in this study. The lack of a prognostic role of the Ki67-LI and HER2 expression in our cohort was most likely caused by the effect of an appropriate therapy in contemporary patients. Patients with HER2 overexpressing tumors are now effectively treated by anti-HER2 antibody drugs and cancers with a high Ki67-LI respond particularly well to cytotoxic chemotherapy [42,43]. In line with our data, studies on more recent cohorts (enrolled between 2002 and 2013) showed that the prognostic relevance of Ki67 was rather limited as compared to its predictive value for response to chemotherapy [42,44]. For HER2, studies involving adequately treated contemporary patients showed either a similar prognosis of both HER2-positive and HER2-negative patients [45] or even a more favorable outcome for HER2-positive patients [46].

TOP2A. TOP2A is another protein that is expressed during the G2/M phase of the cell cycle and is therefore regarded as a proliferation marker [47]. The fact that the subset of tumors with highest TOP2A positivity were mostly HER2-positive is consistent with a coamplification of these genes [48]. The TOP2A gene is known to be located at 17q21, which is only ~700 kb telomeric to HER2 [48,49]. The HER2 amplicon is known to include TOP2A in about 40% of HER2 amplified cases [50]. Several studies had earlier reported associations between a high expression of TOP2A and poor prognosis in breast cancer [49,51]. Irrespective of whether these findings were due to a link with the (prognostic) HER2 amplification or the role of TOP2A as a parameter for high tumor cell proliferation, the lack of a prognostic impact of TOP2A expression in our patients is again consistent with HER2- and/or proliferation-associated treatment effects. The fact that a high TOP2A expression level was also related to aggressive tumor features such as grade and pT stage is consistent, however, with a prognostic role of this parameter in untreated patients.

PD-L1. PD-L1 positivity in tumor cells was also significantly linked to favorable tumor phenotype and prolonged survival in NST carcinomas that were all treated before 2019, when checkpoint inhibitors were approved for use in breast cancer patients in Germany. In line with our data, multiple earlier studies have described associations between tumoral PD-L1 expression and a high density of tumor-infiltrating lymphocytes (TILs) in breast cancer [52,53,54] and in other tumors such as head and neck cancer [55], non-small cell lung cancer [56], and gastric cancer [57] as well as a link between a high density of TILs and favorable prognosis in breast cancer [58] and several other tumor entities [59]. Given the current use of immune checkpoint therapy in the first-line treatment of locally advanced or metastatic PD-L1-positive triple-negative breast carcinoma and a potentially expanding spectrum of therapeutic indications, it seems likely that the prognostic impact of PD-L1 expression on disease outcome in future studies will be different from our findings.

Prognosis score. The successful identification of five parameters that were linked to overall survival enabled us to define multiparametric prognosis scores in this study. Prognostic scores were identified both based on the manually defined prognostic groups in univariate analyses and by using hierarchical clustering in combination with time-dependent receiver operating characteristic curves of the R “riskRegression” 12 package. Although both approaches suffered from limitations such as the lack of a separation in test and training sets, it is notable that our five-parameter score of PR, ER, AR, GATA3, and PD-L1 provided strong prognostic information that was independent of tumor grade, pT, and pN status in multivariate analysis. We consider these observations as evidence for the feasibility of developing robust prognostic tests based on multiplex immunohistochemistry. It is generally accepted that clinically relevant prognostic tests are likely to require the simultaneous analysis of multiple gene products. The currently established test systems such as OncotypeDX, EndoPredict, MammaPrint, Prosigna, Breast Cancer Index, or Mammostrat [10,11,60] are all based on RNA expression analysis which suffers from the variable admixture of multiple components of non-neoplastic tissues which prevents the evaluation of pure tumor tissue [14,15,16,17,18]. It appears highly likely that this shortcoming will be overcome by multiplex immunohistochemistry, especially since methodological advancements enable the parallel analysis of a continuously rising number of antibodies [19,20]. 

Limitations. A limitation of this study is the lack of data on adjuvant therapy of patients as this can have an impact on the prognostic value of some of the investigated markers such as HER2, ER, or PR [46,61]. Moreover, the number of evaluable tumors was limited. Larger cohorts of patients might facilitate the analysis of an increased number of prognosis markers, especially given that the technical progress will enable the analysis of 20 or more prognostic biomarkers simultaneously.

## 5. Conclusions

The data from this study show that automated breast cancer detection in combination with an artificial intelligence-based analysis of multiplex fluorescence immunohistochemistry enables a rapid and reliable analysis of multiple prognostic parameters. The major advantage of this method is the strict limitation of the analysis to malignant cells that cannot be achieved using RNA-based panel analysis.

## Figures and Tables

**Table 1:** Association between biomarkers and breast cancer NST phenotype.

Clinical Parameter	n	PR mfIHC Score	*p*-Value	ER mfIHC Score	*p*-Value	AR mfIHC Score	*p*-Value
pT			<0.0001		0.45		<0.0001
pT1	674	40 (±21)		38 (±30)		46 (±20)	
pT2	570	32 (±23)		39 (±33)		41 (±22)	
pT3-4	116	26 (±24)		35 (±32)		36 (±23)	
pN			<0.0001		0.57		0.0009
pN−	619	38 (±22)		38 (±31)		45 (±20)	
pN+	471	31 (±23)		37 (±31)		41 (±22)	
Metastasis			<0.0001		0.0042		<0.0001
M−	179	41 (±19)		40 (±29)		46 (±17)	
M+	93	20 (±20)		29 (±29)		33 (±24)	
Grade			<0.0001		<0.0001		<0.0001
1	172	47 (±16)		34 (±27)		48 (±15)	
2	733	40 (±21)		43 (±30)		45 (±19)	
3	493	24 (±22)		30 (±32)		37 (±25)	
Clinical parameter	n	GATA3 mfIHC score	*p*-value	TROP2 mfIHC score	*p*-value	HER2 mfIHC score	*p*-value
pT			<0.0001		0.0002		0.9525
pT1	674	67 (±21)		83 (±26)		3 (±13)	
pT2	570	61 (±25)		78 (±30)		3 (±12)	
pT3-4	116	53 (±27)		73 (±31)		3 (±12)	
pN			0.0008		0.13		0.74
pN−	619	65 (±23)		81 (±27)		3 (±12)	
pN+	471	60 (±24)		79 (±28)		4 (±13)	
Metastasis			<0.0001		0.0576		0.29
M−	179	64 (±18)		79 (±29)		5 (±14)	
M+	93	49 (±26)		72 (±34)		3 (±10)	
Grade			<0.0001		<0.0001		<0.0001
1	172	73 (±14)		88 (±20)		1 (±4)	
2	733	68 (±20)		81 (±27)		2 (±10)	
3	493	52 (±28)		74 (±32)		6 (±16)	
Clinical parameter	n	PD-L1 mfIHC score	*p*-value	Ki67 labeling index	*p*-value	TOP2A labeling index	*p*-value
pT			0.11		0.0044		<0.0001
pT1	674	10 (±15)		7 (±10)		1 (±3)	
pT2	570	9 (±14)		9 (±10)		2 (±3)	
pT3-4	116	8 (±17)		9 (±12)		2 (±4)	
pN			0.0001		0.071		0.0002
pN−	619	11 (±16)		7 (±10)		1 (±2)	
pN+	471	7 (±12)		8 (±10)		2 (±3)	
Metastasis			0.10		0.0021		<0.0001
M−	179	7 (±14)		6 (±8)		1 (±2)	
M+	93	5 (±9)		10 (±12)		2 (±3)	
Grade			0.0953		<0.0001		<0.0001
1	172	11 (±14)		5 (±9)		0 (±1)	
2	733	9 (±14)		6 (±8)		1 (±2)	
3	493	10 (±17)		13 (±12)		3 (±4)	

## Data Availability

Data are contained within the article and Supplementary Materials and are available for bona fide researchers who request it from the authors.

## Supplementary Materials

The following supporting information can be downloaded at: https://www.mdpi.com/article/10.3390/biomedicines11123175/s1, Figure S1: Optimal distance for automated breast cancer detection by normal gland exclusion, Figure S2: Quantification of nine prognosis markers, Figure S3: Calculation of the mfiHC Score, Figure S4: Time-dependent receiver operating characteristic (ROC) curves for overall survival 4 years after surgery, Figure S5: Validation of mfIHC expression analysis, Figure S6: Association between prognostic marker expression and clinic-histopathological features, Table S1: Patient characteristics of the TMA cohort, Table S2: List of the used antibodies, antigen retrieval (AR), dilutions, and opal dyes for multiplex fluorescence immunohistochemistry, Table S3: Classification performance of three different approaches for automated breast cancer detection on the validation set (n = 613 glands), Table S4: Classification performance of three DeepLabv3+ convolutional networks for the detection of HER2^+^, ER^+^, and PR^+^ patients on the validation set (n = 356), Table S5: Multivariate analysis of mfIHC scores of the progesterone receptor (PR), estrogen receptor (ER), androgen receptor (PR), GATA3, TROP2, HER2, PD-L1, and the fraction of Ki67 and TOP2A with regards to tumor stage (pT-Stage), nodal stage (N-Stage), and tumor grade (n = 350).
