# Supplementary material for: Automated Prognosis Marker Assessment in Breast Cancers Using BLEACH&STAIN Multiplexed Immunohistochemistry
Source: Biomedicines. 2023 Nov 29;11(12):3175. doi: 10.3390/biomedicines11123175 (PMC10741079; doi:10.3390/biomedicines11123175)
Supplement: Supplementary file 1 [file biomedicines-11-03175-s001.zip › biomedicines-2703957-supplementary.pdf]

Supplementary Materials

Manuscript title: Automated prognosis marker assessment in 1404 breast cancers using artificial intelligence and BLEACH&STAIN multiplexed immunohistochemistry.

Section S1: Supplementary Figures 1-7 2

Figure S1: Optimal distance for automated breast cancer detection by normal gland exclusion. 2

Figure S2: Quantification of 9 prognosis markers. 3

Figure S3: Calculation of the mfiHC Score. 4

Figure S4: Time-dependent receiver operating characteristic (ROC) curves for overall survival 4 years after surgery. 5

Figure S5: Validation of mfIHC expression analysis. 6

Figure S6: Association between prognostic marker expression and clinic-histopathological features. 7

Section S2: Supplementary Tables 1-4 8

Table S1: Patient characteristics of the TMA cohort 8

Table S2: List of the used antibodies, antigen retrieval (AR), dilutions, and Opal dyes for multiplex fluorescence immunohistochemistry. 9

Table S3: Classification performance of three different approaches for automated breast cancer detection on the validation set (n=613 glands) 9

Table S4: Classification performance of three DeepLabv3$^+$ convolutional networks for the detection of HER2$^+$, ER$^+$, and PR$^+$ patients on the validation set (n=356). 10

Table S5: Multivariate analysis of mfIHC scores of progesterone receptor (PR), estrogen receptor (ER), androgen receptor (PR), GATA3, TROP2, HER2, PD-L1 and the fraction of Ki67 and TOP2A with regards to tumor stage (pT-Stage), nodal stage (N-Stage) and tumor grade (n=350). 11

Section S1: Supplementary Figures 1-7

Figure S1: Optimal distance for automated breast cancer detection by normal gland exclusion.

(A) A convolutional neural network (U-Net) for cell detection facilitated an additional distance measurement algorithm. The distance between a PanCK$^+$ epithelial cell and the nearest Myosin$^+$ cells was automatically measured. Visualizations of different thresholds for exclusion of benign gland cells based on the distance to the nearest Myosin$^+$ cell are shown.

(B) The highest accuracy of breast cancer detection was achieved by excluding all epithelial cells at a ≤25μm distance to the nearest Myosin$^+$ cell (benign gland cells). For this purpose, 374 annotations of benign and malignant breast glands were made by a pathologist containing a total of 197,493 healthy and malignant cells.

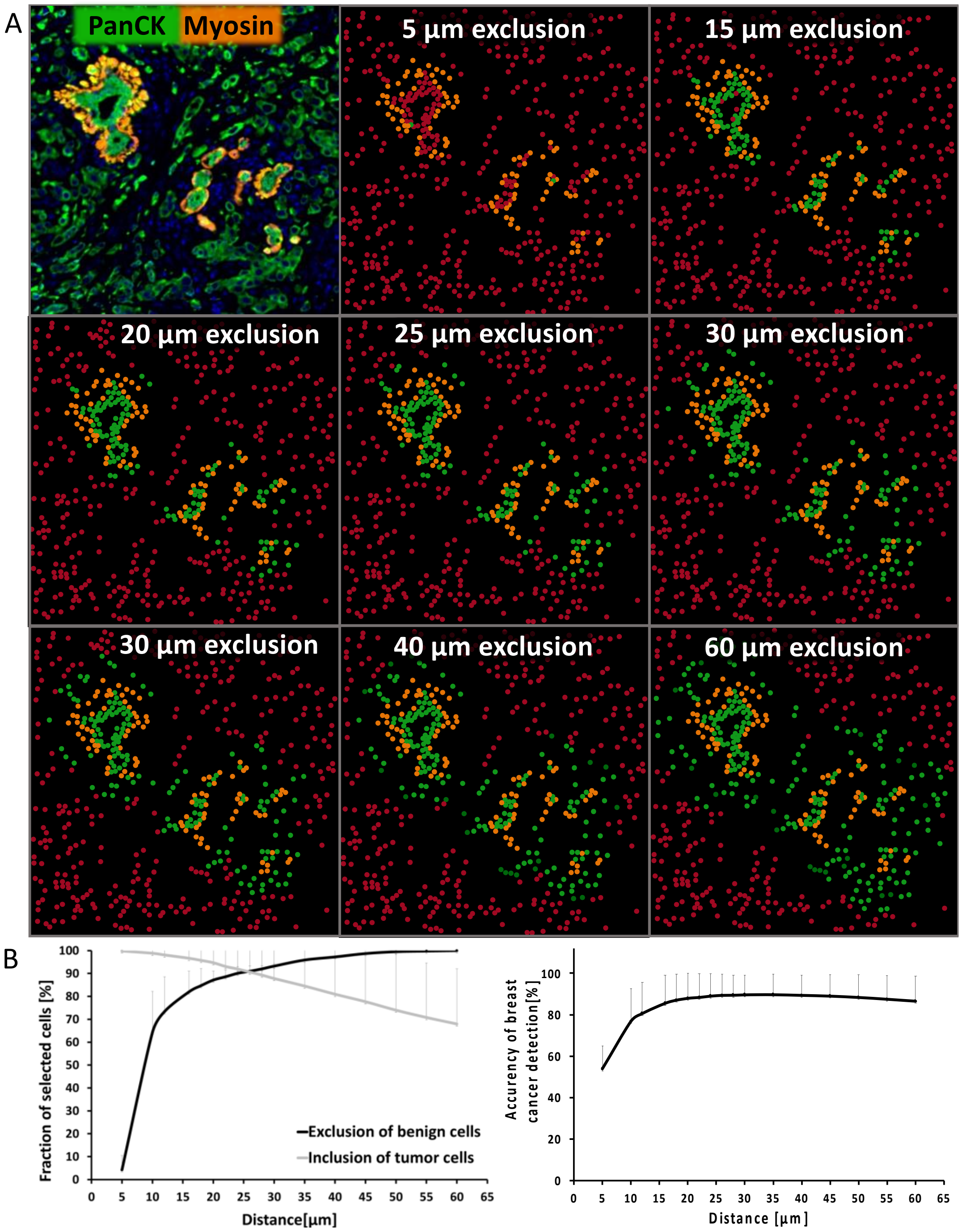

Figure S2: Quantification of 9 prognosis markers.
The proportion of marker positive cells (A), mean marker intensity (B), and the combination of both parameters as mfIHC sum score (C) is shown for each patient. The threshold for each marker in univariate analysis is shown (red: positive; blue: negative).

A Fractions

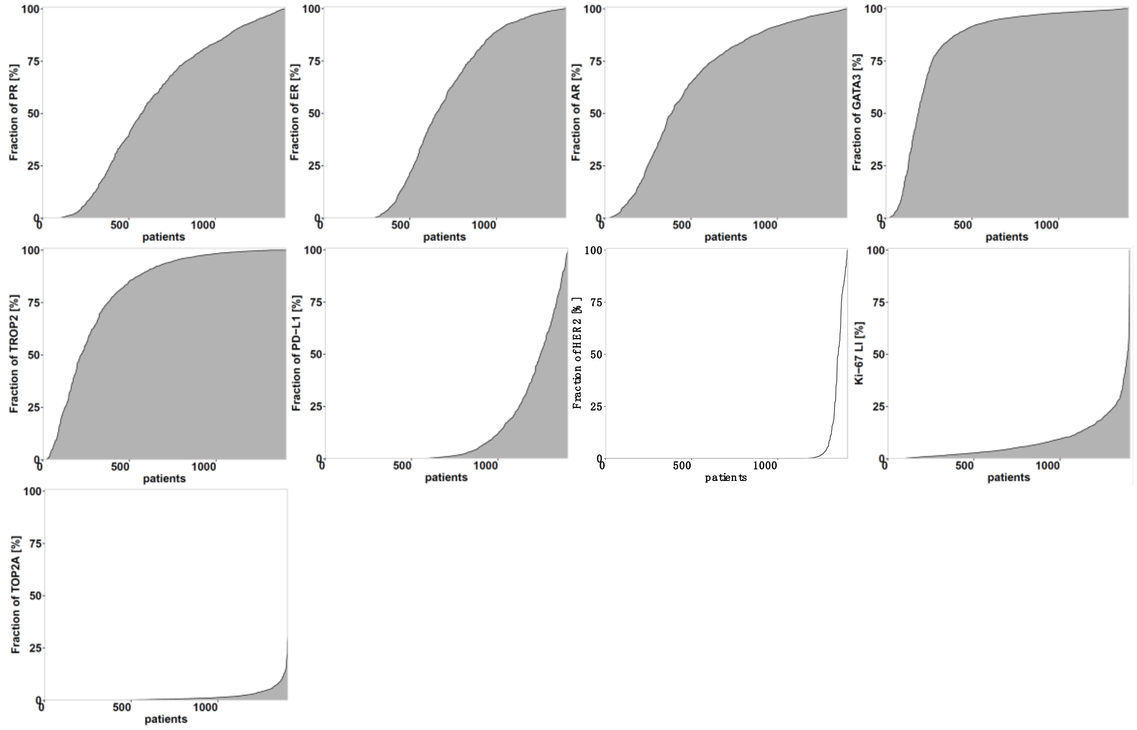

B Intensities

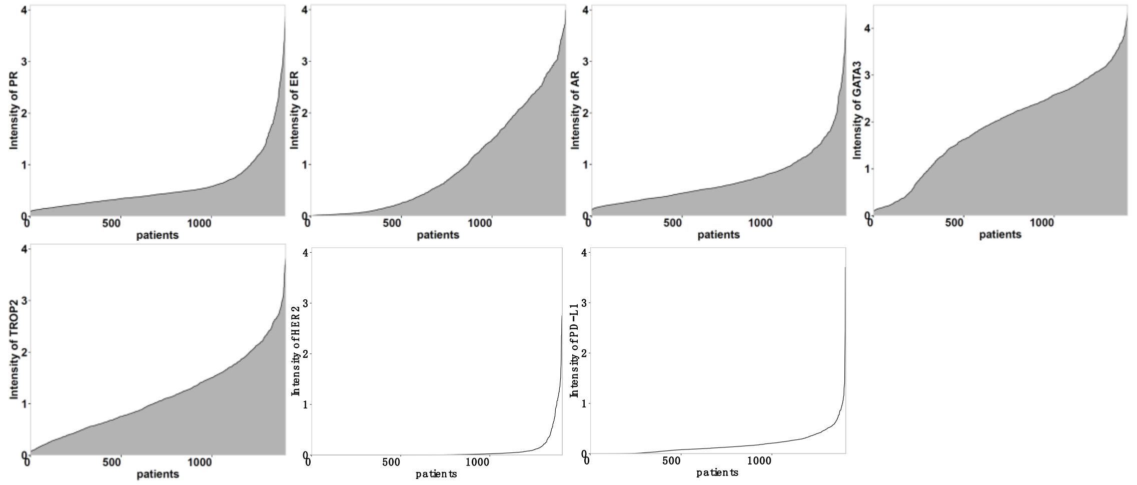

C mfIHC scores

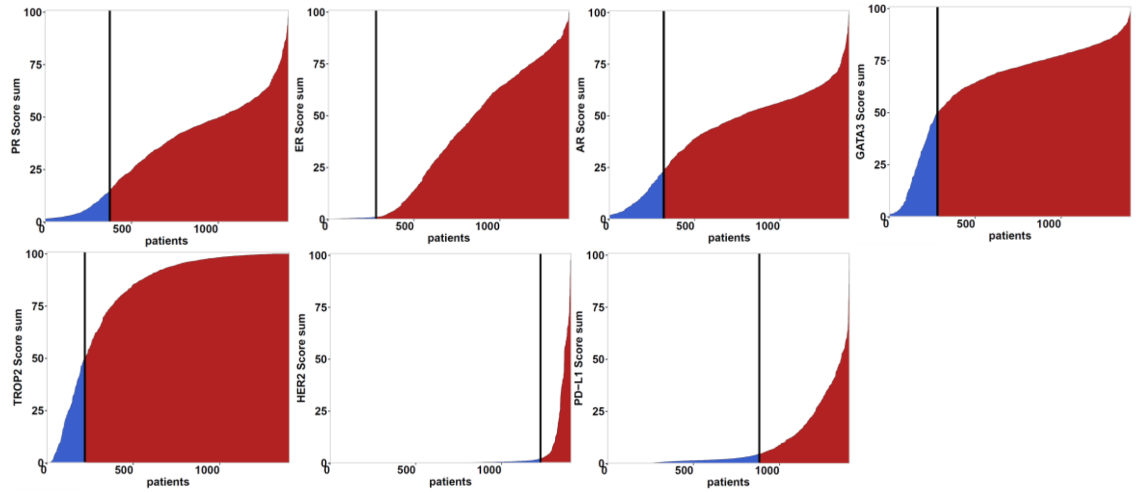

Figure S3: Calculation of the mfiHC Score.

(A)The mfIHC score was calculated by the sum of the min-max normalized (range 0 to 50) proportion of marker positive cells and the min-max normalized (range 0 to 50) mean intensity of marker positive cells for each patient.

(B) Association between the normalized proportion of marker positive cells and the normalized mean marker intensity is shown for each patient.

C) Association between mfIHC score and DeepLabv3^{+} based detection of receptor status.

A

$$mfIHC\ Score = \frac{x - min(x)}{max(x) - min(x)} * 50 + \frac{y - min(y)}{max(y) - min(y)} * 50$$

x = Fraction of marker positive cells
y = Mean mIF marker intensity

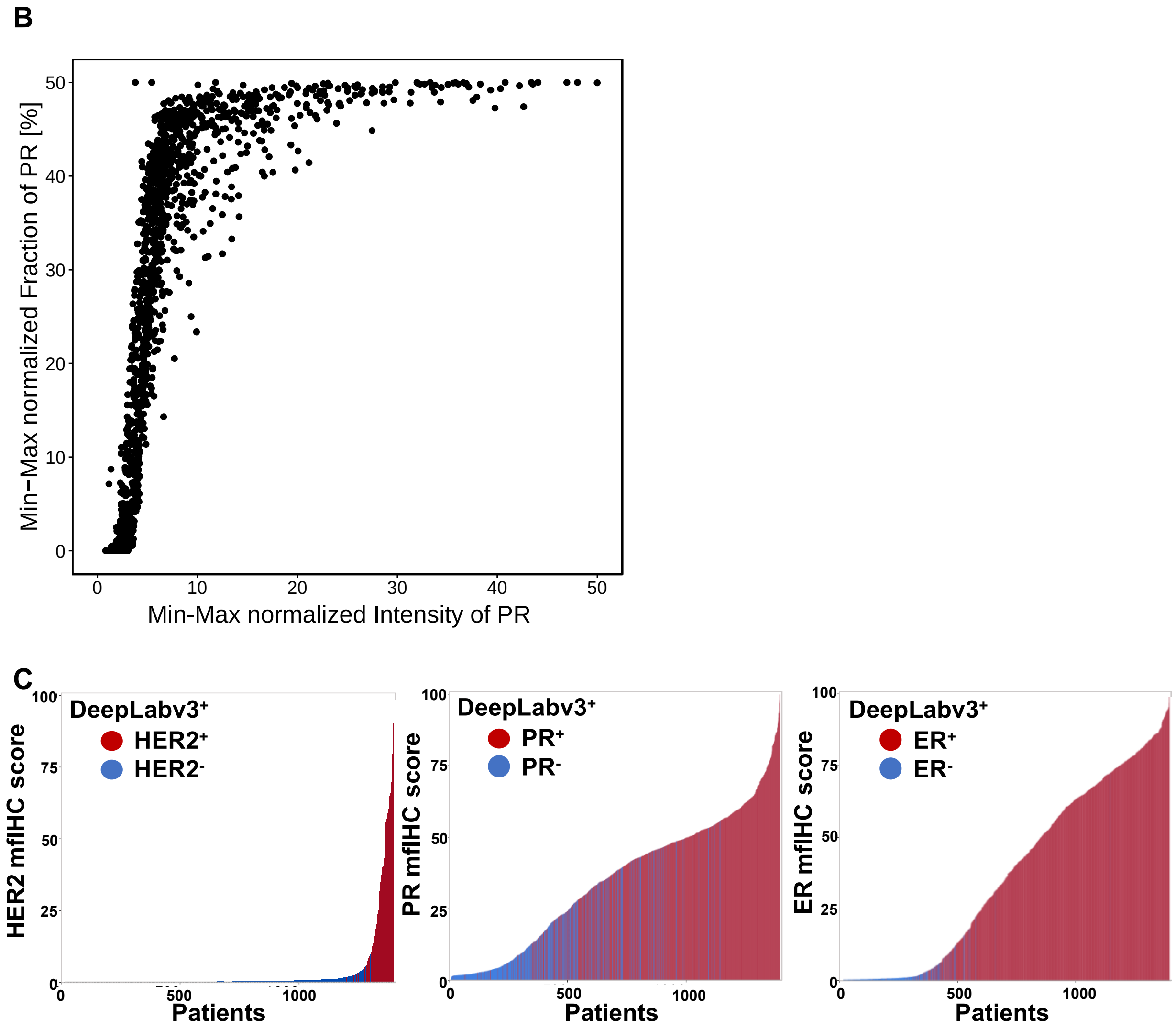

Figure S4: Time-dependent receiver operating characteristic (ROC) curves for overall survival 4 years after surgery.

Shown are time-dependent ROC curves and corresponding area under the curves (AUC) for prognosis marker assessment using no exclusion, distance-based benign gland exclusion and combined DeepLabv3+ tumor detection and distance-based benign gland exclusion.

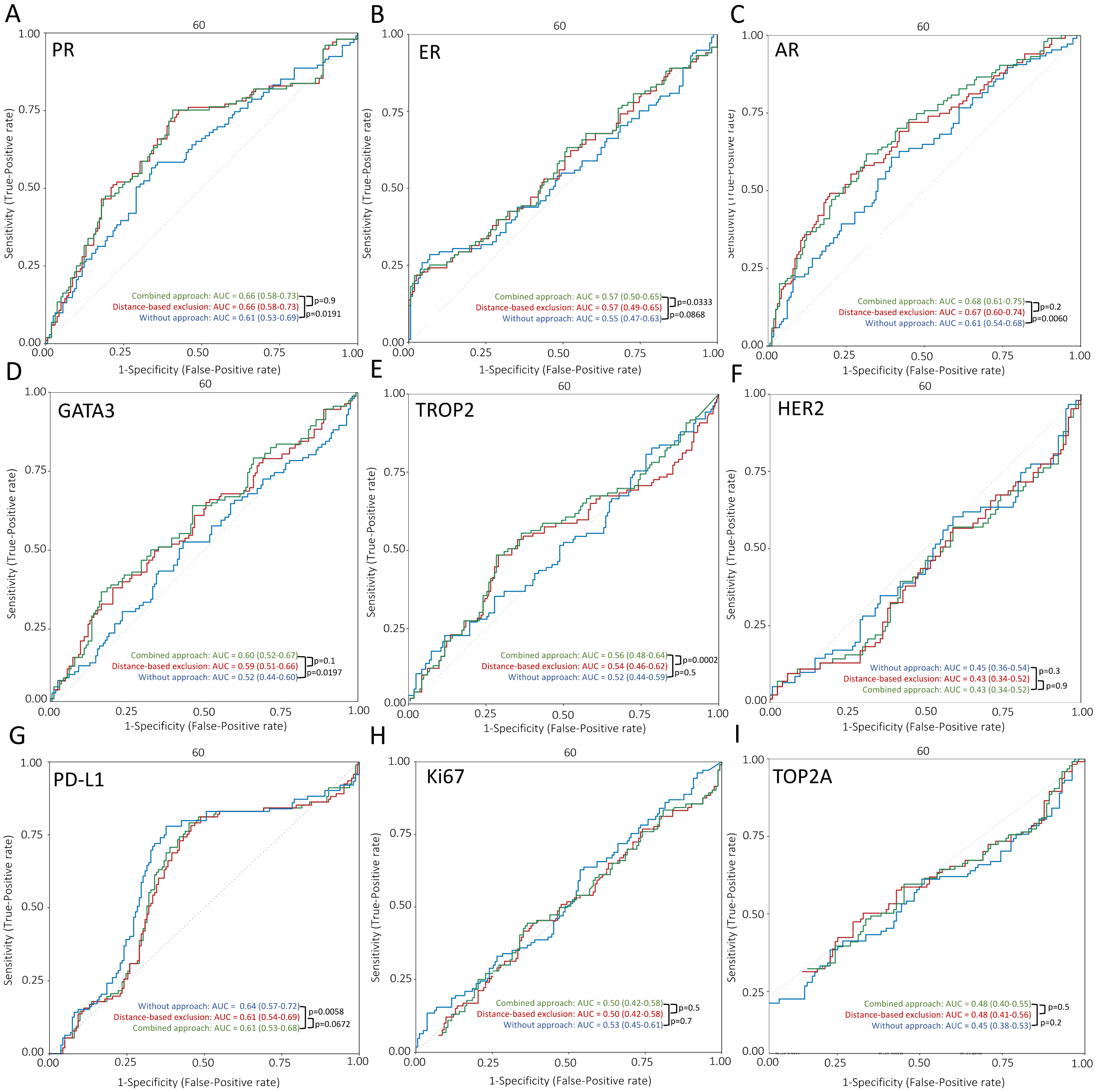

Figure S5: Validation of mfIHC expression analysis.
Multiplex immunofluorescence expression analysis results were highly concordant with results from previous studies using conventional brightfield immunohistochemistry.

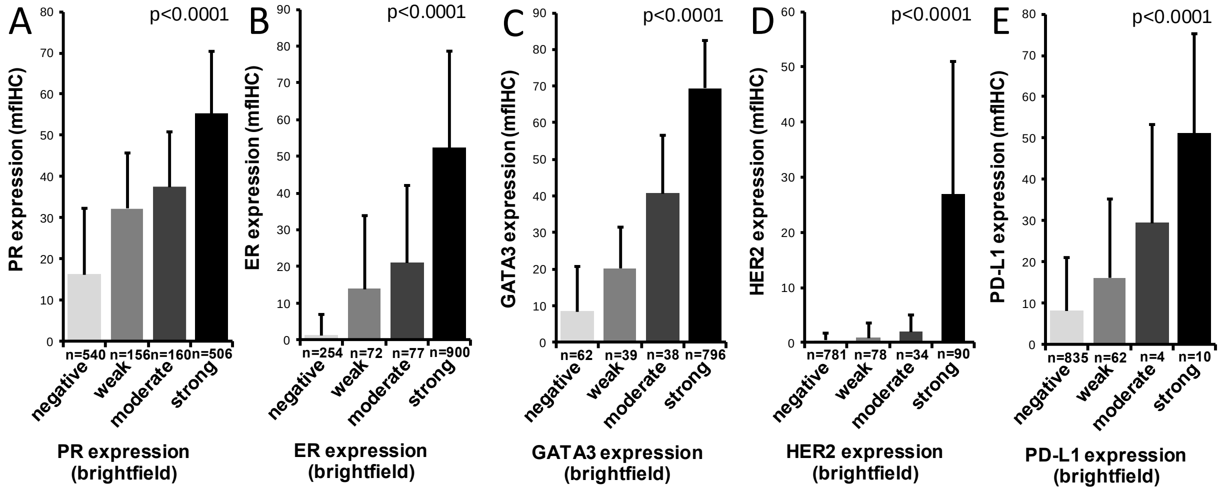

Figure S6: Association between prognostic marker expression and clinico-histopathological features.

The expression of the analyzed prognostic marker and the clinico-histopathological parameters of 1404 breast cancer patients are shown according to the t-distributed stochastic neighbor embedding (t-SNE) algorithm. Each point represents a single patient, and the axes (t-SNE1 and t-SNE2) have arbitrary units. A high similarity between the breast cancer patients is indicated by a close clustering in the t-SNE plot.

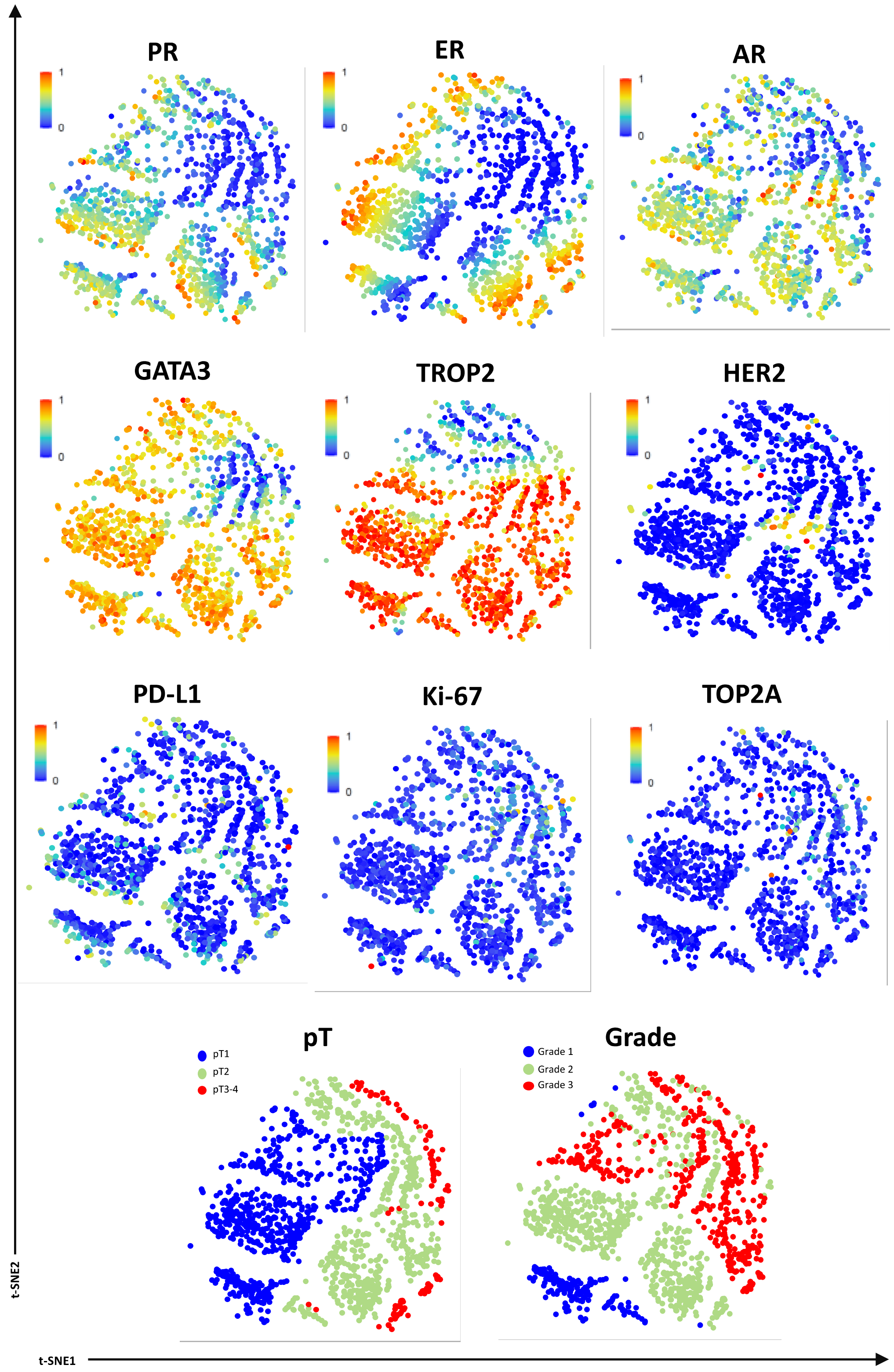

Section S2: Supplementary Tables 1-4

Table S1: Patient characteristics of the TMA cohort

	No. of patients (%)	
**Patients characteristics**	**Total study cohort on TMA (n=1530)**	**Overall death among categories**
Follow-up - no. (%)	627 (41.0)	119 (19.9)
Median - months (95% confidence interval)	49 (46-49)	-
Age (median) - years	67	-
Sex		
Male	6 (0.4)	-
Female	359 (23.5)	-
Missing data	1165 (76.1)	-
pT stage - no. (%)		
pT1	747 (48.8)	26 (8.5)
pT2	604 (39.5)	56 (22.2)
pT3-4	124 (8.1)	28 (44.4)
Missing data	55 (3.6)	-
pN stage - no. (%)		
pN-	677 (44.2)	35 (19.8)
pN+	508 (33.2)	58 (26.4)
Missing data	345 (22.5)	-
M status - no. (%)		
M-	196 (12.8)	18 (9.6)
M+	105 (6.9)	60 (67.4)
Missing data	1229 (80.3)	-
Grade - no. (%)		
1	192 (12.5)	6 (8.5)
2	796 (52.0)	63 (20.0)
3	534 (34.9)	51 (19.5)
Missing data	8 (0.5)	-

Table S2: List of the used antibodies, antigen retrieval (AR), dilutions, and Opal dyes for multiplex fluorescence immunohistochemistry.

Antibody target	Identifier	AR (pH value)	Dilution	Staining position	Opal dye	BLEACH&STAIN
GATA3	MSVA, Clone: MSVA-450M<br>Cat#: 2980-450M	7.8	1:15	1	520	Cycle 1
HER2	DAKO, Clone: polyclonal<br>Cat#: SK001	7.8	1:9	2	570	Cycle 1
PD-L1	MSVA, Clone: MSVA-711R<br>Cat#: 2083-711R	7.8	1:450	3	650	Cycle 1
PR	MSVA, Clone: MSVA-570R<br>Cat#: 3332-570R	9.0	1:5	4	520	Cycle 2
AR	MSVA, Clone: MSVA-367R<br>Cat#: 2145-367R	9.0	1:50	5	570	Cycle 2
ER	MSVA, Clone: MSVA-564R<br>Cat#: 4670-564R	9.0	1:20	6	650	Cycle 2
TROP2	MSVA, Clone: MSVA-733R<br>Cat#: 3648-733R	9.0	1:150	7	520	Cycle 3
TOP2A	MSVA, Clone: MSVA-802R<br>Cat#: 3172-802R	9.0	1:150	8	570	Cycle 3
Myosin	MSVA, Clone: MSVA-375R<br>Cat#: 2450-375R	9.0	1:50	9	650	Cycle 3
PanCK	MSVA, Clone: MSVA-000R<br>Cat#: 2105-000R	9.0	1:300	10	520	Cycle 4
Ki67	MSVA, Clone: MSVA-267M<br>Cat#: 2082-267M-001	9.0	1:50	11	570	Cycle 4

(MSVA: MS Validated Antibodies GmbH, AR: antigen retrieval)

Table S3: Classification performance of three different approaches for automated breast cancer detection on the validation set (n=613 glands)

Approach	Healthy breast tissue	Breast cancer	Sensitivity (95% CI)	Specificity (95% CI)	Accuracy (95% CI)
	number of glands			*percent*	
Cell-AI & Distance exclusion	364	249	92.1<br>(87.6 - 96.5)	94.9<br>(91.1 - 98.7)	95.4<br>(93.4 - 97.4)
Cell-AI & Tumor detection DeepLabv3^{+}	364	249	97.4<br>(95.9 - 98.9)	98.5<br>(97.1 - 99.9)	98.4<br>(97.6 - 99.3)
Combined approach	364	249	96.9<br>(95.3 - 98.4)	98.6<br>(97.2 - 100.0)	98.4<br>(97.4 - 99.3)

Table S4: Classification performance of three DeepLabv3$^+$ convolutional networks for the detection of HER2$^+$, ER$^+$, and PR$^+$ patients on the validation set (n=356).

Approach	Receptor positive	Receptor negative	Sensitivity (95% CI)	Specificity (95% CI)	Accuracy (95% CI)
	number of patients			*percent*	
HER2 DeepLabv3$^+$	42	314	90.4 (90.2 - 90.5)	97.5 (97.4 - 97.5)	96.6 (96.6 - 96.7)
ER DeepLabv3$^+$	255	101	95.3 (95.2 - 95.4)	99.0 (99.0 - 99.1)	96.4 (96.3 - 96.4)
PR DeepLabv3$^+$	251	105	95.2 (95.2 - 95.3)	99.1 (99.0 - 99.1)	96.4 (96.3 - 96.4)

Table S5: Multivariate analysis of mfIHC scores of progesterone receptor (PR), estrogen receptor (ER), androgen receptor (PR), GATA3, TROP2, HER2, PD-L1 and the fraction of Ki67 and TOP2A with regards to tumor stage (pT-Stage), nodal stage (N-Stage) and tumor grade (n=350).

Tumor subset<br>Analyzable (N)	Progesteron receptor (PR)<br>HR (95% CI) p-value<br>350	p-value	Estrogen receptor (ER)<br>HR (95% CI) p-value<br>350	p-value	Androgen receptor (AR)<br>HR (95% CI) p-value<br>350	p-value
**pT-Stage**<br>T2 vs. T1<br>T3-4 vs. T2	**2.45** (1.40-4.30) p=0.0017<br>**1.79** (1.03-3.12) p=0.0406	**p<0.0001**	**2.59** (1.48-4.52) p=0.0008<br>**1.94** (1.12-3.37) p=0.0180	**p<0.0001**	**2.55** (1.47-4.45) p=0.0009<br>**1.83** (1.05-3.20) p=0.032	**p<0.0001**
**N-Stage**<br>N+ vs. N0	**1.26** (0.78-2.04)	**p=0.3**	**1.29** (0.80-2.08)	**p=0.3**	**1.31** (0.81-2.10)	**p=0.3**
**Grade**<br>2 vs. 1<br>3 vs. 2	**1.69** (0.59-4.34) p=0.3314<br>**0.87** (0.54-1.40) p=0.5669	**p=0.6**	**1.73** (0.60-4.94) p=0.3089<br>**0.91** (0.56-1.48) p=0.6907	**p=0.6**	**1.68** (0.59-4.82) p=0.3316<br>**0.93** (0.59-1.49) p=0.7698	**p=0.6**
**Grouping for marker analysis**	>15 vs. 0-15		>1 vs. <1		>23 vs. <23	
**Marker risk ratios**	**0.55** (0.34-0.89)	**p=0.0149**	**0.62** (0.37-1.05)	**p=0.1**	**0.54** (0.33-0.89)	**p=0.0151**
**Tumor subset**<br>**Analyzable (N)**	**GATA3**<br>**HR (95% CI) p-value**<br>350	**p-value**	**TROP2**<br>**HR (95% CI) p-value**<br>350	**p-value**	**HER2**<br>**HR (95% CI) p-value**<br>350	**p-value**
**pT-Stage**<br>T2 vs. T1<br>T3-4 vs. T2	**2.59** (1.49-4.53) p=0.0008<br>**1.83** (1.05-3.19) p=0.0338	**p<0.0001**	**2.63** (1.50-4.59) p=0.0007<br>**1.90** (1.08-3.34) p=0.0256	**p<0.0001**	**2.67** (1.52-4.68) p=0.0006<br>**1.97** (1.13-3.43) p=0.0164	**p<0.0001**
**N-Stage**<br>N+ vs. N0	**1.31** (0.82-2.12)	**p=0.3**	**1.31** (0.81-2.11)	**p=0.3**	**1.34** (0.83-2.16)	**p=0.2**
**Grade**<br>2 vs. 1<br>3 vs. 2	**1.70** (0.59-4.82) p=0.3214<br>**0.96** (0.60-1.52) p=0.8511	**p=0.6**	**1.70** (0.59-4.89) p=0.3270<br>**1.04** (0.66-1.64) p=0.8531	**p=0.6**	**1.70** (0.59-4.88) p=0.3256<br>**1.04** (0.66-1.64) p=0.8607	**p=0.6**
**Grouping for marker analysis**	>50 vs. <50		>50 vs. <50		>2 vs. <2	
**Marker risk ratios**	**0.62** (0.38-1.01)	**p<0.06**	**0.89** (0.51-1.55)	**p=0.7**	**1.18** (0.67-2.07)	**p=0.6**
**Tumor subset**<br>**Analyzable (N)**	**PD-L1**<br>**HR (95% CI) p-value**<br>350	**p-value**	**Ki-67**<br>**HR (95% CI) p-value**<br>350	**p-value**	**TOP2A**<br>**HR (95% CI) p-value**<br>350	**p-value**
**pT-Stage**<br>T2 vs. T1<br>T3-4 vs. T2	**2.55** (1.46-4.45) p=0.0010<br>**1.88** (1.08-3.27) p=0.0262	**p<0.0001**	**2.63** (1.51-4.59) p=0.0007<br>**1.92** (1.11-3.35) p=0.0207	**p<0.0001**	**2.53** (1.44-4.46) p=0.0013<br>**1.99** (1.14-3.48) p=0.0156	**p<0.0001**
**N-Stage**<br>N+ vs. N0	**1.30** (0.80-2.09) p=0.2874	**p=0.3**	**1.32** (0.82-2.13)	**p=0.3**	**1.32** (0.82-2.13)	**p=0.2**
**Grade**<br>2 vs. 1<br>3 vs. 2	**1.59** (0.55-4.59) p=0.3878<br>**1.07** (0.68-1.69) p=0.7627	**p=0.6**	**1.77** (0.61-5.09) p=0.2930<br>**1.08** (0.67-1.72) p=0.7615	**p=0.5**	**1.67** (0.58-4.84) p=0.3408<br>**1.01** (0.63-1.62) p=0.9582	**p=0.6**
**Grouping for marker analysis**	>4.5 vs. <4.5		≥2 vs. <2		≥1 vs. <1	
**Marker risk ratios**	**0.60** (0.31-1.13)	**p=0.1**	**0.90** (0.55-1.47)	**p=0.7**	**1.15** (0.72-1.84)	**p=0.6**
